# Tissue-specific extracellular matrix accelerates the formation of neural networks and communities in a neuron-glia co-culture on a multi-electrode array

**DOI:** 10.1038/s41598-019-40128-1

**Published:** 2019-03-11

**Authors:** Doris Lam, Heather A. Enright, Jose Cadena, Sandra K. G. Peters, Ana Paula Sales, Joanne J. Osburn, David A. Soscia, Kristen S. Kulp, Elizabeth K. Wheeler, Nicholas O. Fischer

**Affiliations:** 1 Physical and Life Sciences Directorate, Lawrence Livermore National Laboratory, Livermore, CA USA; 2 Engineering Directorate, Lawrence Livermore National Laboratory, Livermore, CA USA

## Abstract

The brain’s extracellular matrix (ECM) is a macromolecular network composed of glycosaminoglycans, proteoglycans, glycoproteins, and fibrous proteins. *In vitro* studies often use purified ECM proteins for cell culture coatings, however these may not represent the molecular complexity and heterogeneity of the brain’s ECM. To address this, we compared neural network activity (over 30 days *in vitro*) from primary neurons co-cultured with glia grown on ECM coatings from decellularized brain tissue (bECM) or MaxGel, a non-tissue-specific ECM. Cells were grown on a multi-electrode array (MEA) to enable noninvasive long-term interrogation of neuronal networks. In general, the presence of ECM accelerated the formation of networks without affecting the inherent network properties. However, specific features of network activity were dependent on the type of ECM: bECM enhanced network activity over a greater region of the MEA whereas MaxGel increased network burst rate associated with robust synaptophysin expression. These differences in network activity were not attributable to cellular composition, glial proliferation, or astrocyte phenotypes, which remained constant across experimental conditions. Collectively, the addition of ECM to neuronal cultures represents a reliable method to accelerate the development of mature neuronal networks, providing a means to enhance throughput for routine evaluation of neurotoxins and novel therapeutics.

## Introduction

The brain’s extracellular matrix (ECM) is a macromolecular network composed of proteins and polysaccharides that occupies the space in between neurons and glia, and accounts for approximately 20% of the total volume in the adult brain^1^. Synthesized by both neurons and glial cells, the brain’s ECM is primarily composed of glycosaminoglycans (e.g., hyaluronan), proteoglycans (e.g., neurocan, brevican, versican and aggrecan), glycoproteins (e.g., tenascin-R), and low levels of fibrous proteins (e.g. collagen, fibronectin, and vitronectin)^2–4^. Structurally, the ECM acts as a physical barrier to reduce the diffusion of soluble and membrane-associated molecules and cell migration. It also functions to regulate a number of fundamental neural processes during brain development and can play a role in physiological and pathological conditions in the adult brain, including neurite outgrowth, synaptogenesis, synaptic stabilization, and injury-related plasticity^3,4^.

Typically, *in vitro* studies use a single purified ECM protein type for cell culture coatings, while very few use more than a handful^5^. However, it is important to recognize that reproducing the molecular complexity and heterogeneity of the ECM in the brain may increase the relevance of *in vitro* studies^6^. In recent years, tissue-specific biological scaffolds (e.g., central nervous and cardiovascular systems), which retain the molecular complexity of the ECM, have been prepared by isolating native ECM from decellularized tissue. These native ECM scaffolds have been used for transplantation into animal models for repair and reconstruction of the tissue, and for *in vitro* experimental models to better mimic the *in vivo*-like microenvironment and examine tissue-specific effects on cellular behavior^7,8^. Methods to decellularize brain tissue have been demonstrated for both porcine and rodent brains, and isolated native brain ECM has been used as a coating for two-dimensional (2D) cell culture^7,9–11^, or to serve as biochemical cues for cells encapsulated in three-dimensional (3D) hydrogels^9,11–14^. In 2D cultures, the native brain ECM molecules promoted extensive neurite outgrowth and increased neuronal viability^7,9^. In 3D, it has been shown to support the viability of neurons and neural stem cells (from mice and human), aid in the differentiation of human neural stem cells into neurons, and increase axonal length, ingrowth, and dense network formation in the hydrogel^9,11,13,14^. While the above-mentioned studies show the biocompatibility of native brain ECM and neurons, it is not clear whether glial cells are reactive to the decellularized tissue. Glial reactivity is a response that occurs following injury, inflammation or infection, and *in vitro* could have maladaptive effects on neuronal activity^15^. Moreover, functional characterization of neurons and glial cells in an *in vivo*-like microenvironment is not well studied.

Multi-electrode arrays (MEA) serve as a tool to study the electrical activity of cultured neurons and the networks that form and mature in a 2D *in vitro* model^16–20^. They are now being routinely used to evaluate the effects of chemical and therapeutic compounds on neuronal activity^21,22^. To advance the widespread adoption of this modality, the field is moving towards correlative validation of responses between *in vitro* and *in vivo* systems^23^ while striving to accurately recapitulate *in vivo* morphology, physiology, and function. More sophisticated *in vitro* systems have been established by incorporating different brain regions (or neuronal cells)^24,25^, 3D architecture^9,11,13,14^, specific glial support cells^26,27^, or extracellular matrices^7,9–11^, to more accurately mirror *in vivo* neuronal function and provide more relevant data in response to therapeutics or toxins. In the present study, we increased the compositional relevance of neuronal cultures to include brain tissue-specific and non-specific ECM and examined its effects on long-term co-cultures of neurons and glial cells by comparing neural networks formed on three coating conditions: (1) brain tissue-specific ECM; (2) MaxGel, a commercially-available, non-tissue specific ECM; and (3) poly-D-lysine (PDL), a non-ECM polymeric substrate that enhances cell attachment to microfluidic chips^28^. Cells were grown on a MEA for long-term interrogation of neuronal networks. Flow cytometry was used to quantify the neuronal and glial populations, as well as subpopulations of astrocyte phenotypes in our co-culture model. Immunocytochemistry was used to quantify the proliferative capacity of glial cells, and synaptophysin expression in the neuronal population. Evaluating whether brain tissue-specific (or non-specific) ECM coating improves neuronal network formation and function will be important for developing an *in vitro* model that is more relevant to the *in vivo* brain and can be used for evaluating potential neurotoxins and novel therapeutics.

## Results

### Characterization of biological extracellular matrix molecules (ECM) extracted from rat brain tissue versus human basement membrane

**Figure 1:**
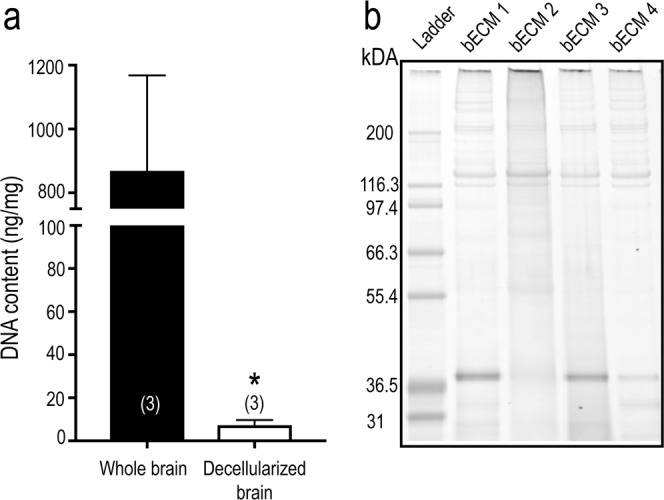
Characterization of decellularized postnatal rat brain ECM. (**a**) The decellularization protocol (see Methods) removed ~99.2% of the DNA content from the brain tissue versus a whole brain. (**b**) SDS-PAGE elucidated the electrophoretic mobility and banding pattern of decellularized bECM favoring high molecular weight proteins, and the reproducibility of the decellularization process on the postnatal rat brain tissue across 4 litters (n = 3 brains/litter). Black box indicates cropped gel. Full-length gel, including MaxGel samples, is presented in Supplementary Fig. 1. Results were analyzed using unpaired t-test, with statistical significance at a level of *p < 0.05.

Brain tissue-specific ECM (bECM) was extracted from decellularized rat brain tissue using a protocol previously described (see Methods)^12^. Before the extracted bECM was used for cell culture experiments, we compared the DNA content of the decellularized brain tissue to unprocessed whole brain tissue. Quantification of double-stranded (ds) DNA content confirmed that 99.2% of DNA in the purified bECM was removed during the decellularization process (Fig. 1a). MaxGel, a commercially available, non-specific ECM, was chosen for comparison. MaxGel is derived from human basement membrane extract and contains collagen, laminin, fibronectin, tenacin, elastin and a number of proteoglycans and glycosaminoglycans^29^. The dsDNA level of our bECM (7.3 ± 2.4 ng/mg (n = 3)) was comparable to MaxGel (0.20 ± 0.02 ng/mg (n = 2)) (data not shown). The solubilized protein profiles were qualitatively compared between matrices using protein gel electrophoresis at comparable ECM concentrations (Fig. 1b) and demonstrated relatively reproducible constituents across four independent preparations. Consistent with previous studies^13,14^, the bECM matrix showed distinct banding patterns, in terms of both molecular weight and band intensity, and was enriched for high molecular weight proteins (>116 kDa). Previous studies of decellularized rat and porcine brain showed that the soluble proteins extracted included elastin, sulfated glycosaminoglycans, myelin, collagen I, collagen III, collagen IV, and laminin^9–11^, as well as low levels of the growth factors VEGF, FGF, BDNF and NGF^10,30^. The bECM prepared using an alternative protocol (originally used to prepare decellularized porcine brain tissue^14^) also exhibited a similar banding pattern to the decellularized protocol for rat brain tissue (data not shown). The preferential banding patterns for high molecular weight proteins were distinct from MaxGel ECM, which displayed a variety of proteins bands spanning a wide range of molecular weights (Supplementary Fig. 1).

### Functional characterization of neural networks formed on brain tissue-specific and non-specific ECM coating

**Figure 2:**
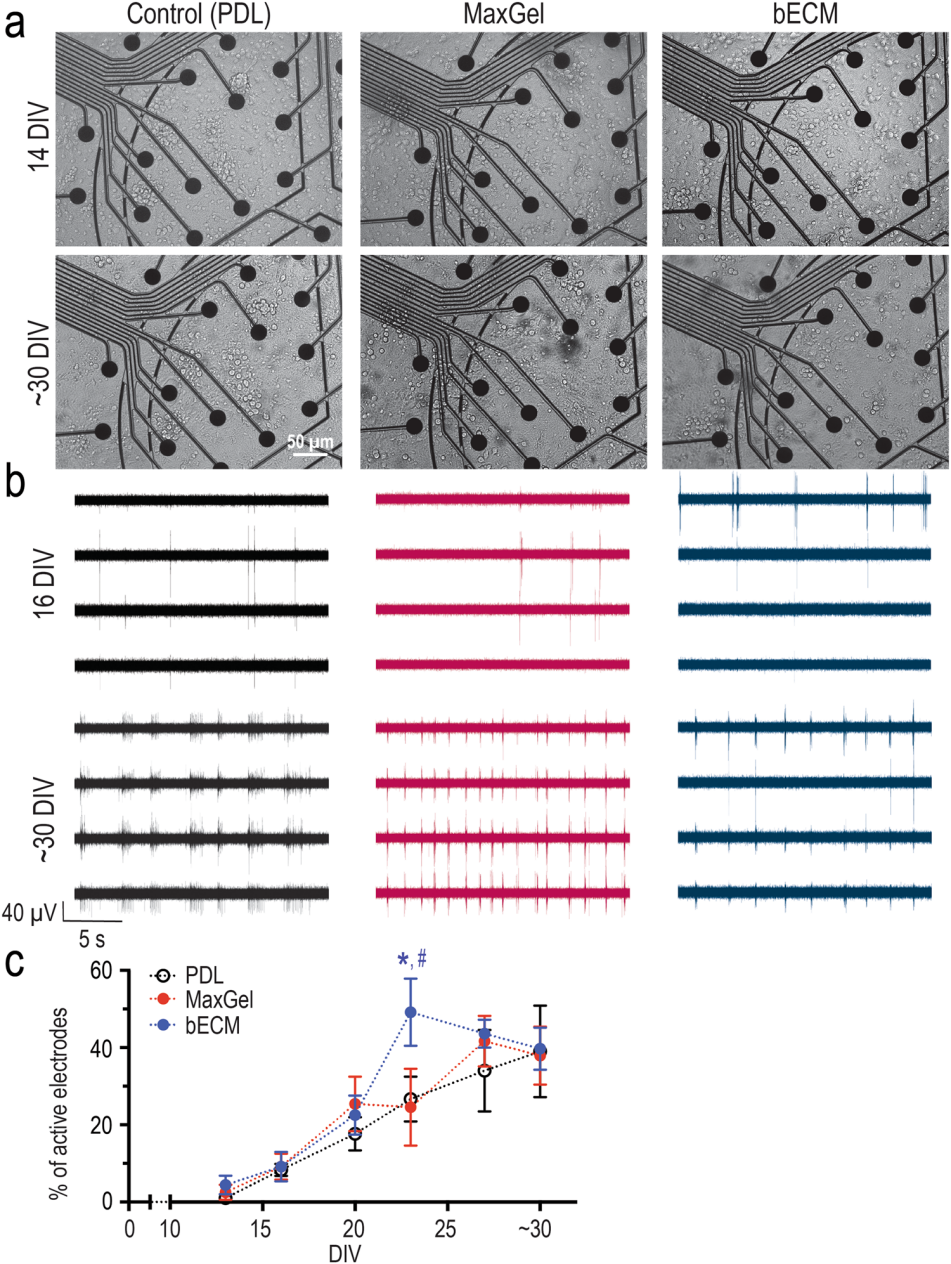
Biocompatibility of ECM coatings for long-term neuron-glia co-cultures. (**a**) Brightfield microscopy was used to monitor neurons co-cultured with glia grown on MEA devices (and control plates, not shown) in the absence (control, left panels) and presence of ECM coatings, MaxGel (middle panels) or bECM (right panels). Scale bar = 50 μm. (**b**) Representative traces of neuronal activity observed at 16 and ~30 DIV. (**c**) Line graph summarizes the number of active electrodes across ~30 DIV. Data are mean ± SEM for the number of devices in control (n = 5), MaxGel (n = 4) and bECM (n = 6) and were analyzed using repeated measures two-way ANOVA with Tukey’s post hoc test. Statistical significances are indicated between PDL and bECM (*) and bECM and MaxGel (^#^) at a significance level of *p* < 0.05.

To evaluate the morphological and electrophysiological development of our networks, primary embryonic cortical rat neurons were grown on both custom MEA devices fabricated in-house^23,25^ and in control 96-well plates for 30–32 (denoted as ‘~30’ hereafter) days *in vitro* (DIV). The two ECM coatings used in this study (bECM and MaxGel) were compared to PDL, a synthetic non-ECM coating molecule used to enhance cell attachment to surfaces via electrostatic interactions (control group)^28^. Since astrocytes or astrocyte-derived molecules improve synapse formation, maturation, and support electrophysiological activity *in vitro*^26,31–33^, the neuronal cultures were not treated with an anti-mitotic agent. This permits the growth of glial cells that are naturally present in most preparations of purified neurons. Cell morphology and neural activity were monitored over the course of ~30 DIV on MEA devices (Supplementary Fig. 2). Representative brightfield images at 14 and ~30 DIV show the distribution of cells across a section of the MEA device (Fig. 2a). Representative traces from active electrodes exhibiting neural activity (i.e., action potential spikes) for these cultures are shown in Fig. 2b. Each electrode is surrounded by multiple cells (Fig. 2a) and can record activity from either single or multiple established neural networks. The onset of neural activity was variable across devices within each experimental condition. While neural activity was detectable by 8 DIV, this activity was scarce and sporadic: 40% of MEA devices from the PDL group (n = 2/5), 50% of devices in MaxGel group (n = 2/4), and 33% of devices from the bECM group (n = 2/6) showed signs of activity (data not shown). At 13 DIV, 100% of devices coated with ECM exhibited neural activity (i.e., n = 4/4 for MaxGel, and n = 6/6 for bECM), compared to 40% of devices in the PDL group (n = 2/5). By 16 DIV all devices exhibited spontaneous neural activity. At a network level, the percentage of active electrodes exhibiting spiking activity increased over time in culture (Fig. 2c). By 23 DIV, at least 50% of the electrodes on devices coated with bECM showed activity, which was significantly higher than both MaxGel (~25%, p < 0.05) and PDL (~27%, p < 0.05) groups (Fig. 2c). However, by ~30 DIV, no statistical differences for the number of active electrodes among the groups were observed. Representative raster plots are shown at 16, 23 and ~30 DIV, illustrating the timing of neuronal spikes (each hash mark equates to a spike) over a period of 10 minutes (Supplementary Figs. 4–6). In particular, the number of active electrodes and the changes in spiking activity both increased over time for each experimental condition: PDL (Supplementary Fig. 4a), MaxGel (Supplementary Fig. 5a), and bECM (Supplementary Fig. 6a).

**Figure 3:**
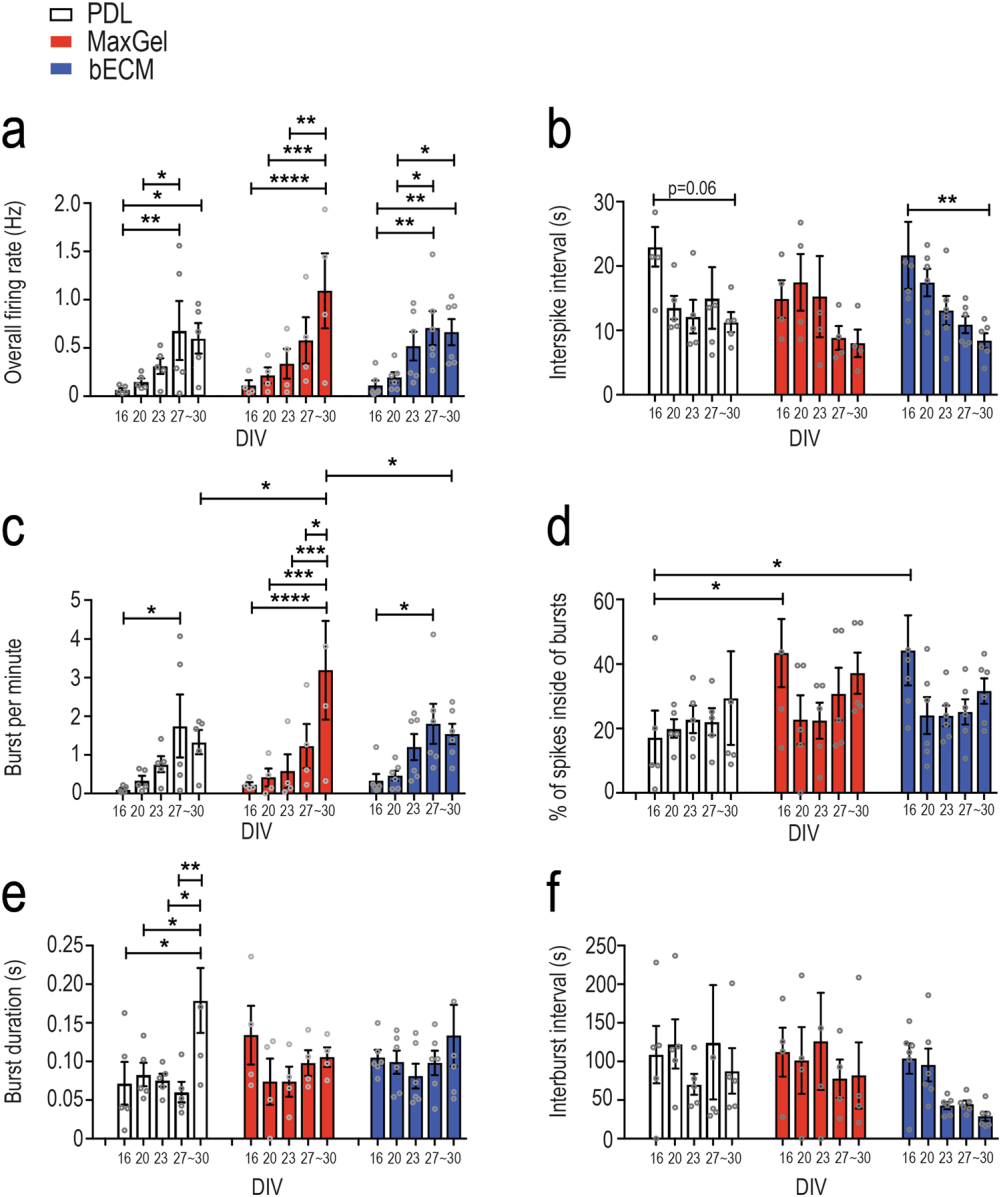
Features of neural network activity for co-cultures grown in the presence and absence of an ECM coating. Bar graph summarizes each electrophysiology feature across a period of ~30 DIV for each experimental condition: PDL (white), MaxGel (red), and bECM (blue). Features of neural activity include: overall firing rate (**a**), interspike interval (**b**), burst per minute (**c**), percentage of spikes inside of bursts (**d**), burst duration (**e**) and interburst interval (**f**). Individual data points are represented as open circles, and summary data bars are presented as mean ± SEM for the number of devices in PDL (n = 5), MaxGel (n = 4) and bECM (n = 6). Data were analyzed using repeated measures two-way ANOVA with Tukey’s post hoc test. Statistical significances are at a level of *p < 0.05, **p < 0.01, ***p < 0.001, and ****p < 0.0001.

Data obtained after 16 DIV were used to compare specific features of neuronal activity across experimental conditions, as this was the time when all devices, in all groups, demonstrated neural activity. Feature analysis of neural activity included single action potential spikes and bursts, which are episodes of relatively fast spikes that are separated by periods of quiescence. Across all groups, the overall firing rate (i.e., number of spikes over the 10-minute recording) increased over days in culture (Fig. 3a), which paralleled the decrease in interspike interval, a feature that was pronounced in the bECM group at ~30 DIV (p < 0.01) compared to PDL (p = 0.06) (Fig. 3b). For bursting features (defining parameters specified in Methods), increases in burst rate (i.e., number of bursts per minute over the 10-minute recording) were found over DIV for all groups (Fig. 3c), suggesting that the increase in overall firing rate (Fig. 3a) was attributed to bursting activity. However, the burst rate of neurons cultured on MaxGel (3.19 ± 1.28  bursts per minute) doubled those on PDL (1.33 ± 0.3, p < 0.05) and bECM (1.54 ± 0.26, p < 0.05) by ~30 DIV. In the presence of an ECM coating (MaxGel or bECM) at 16 DIV, significantly more spikes in a burst were observed (p < 0.05) compared to PDL devices, despite showing comparable durations in bursting activity across all groups at that same time point. While burst duration remained stable across ~30 DIV in ECM coating conditions, the burst duration had doubled in the PDL group (Fig. 3e). Additionally, while not significant, interburst interval for the bECM group decreased over time; whereas both PDL and MaxGel groups remained consistent (Fig. 3f).

**Figure 4:**
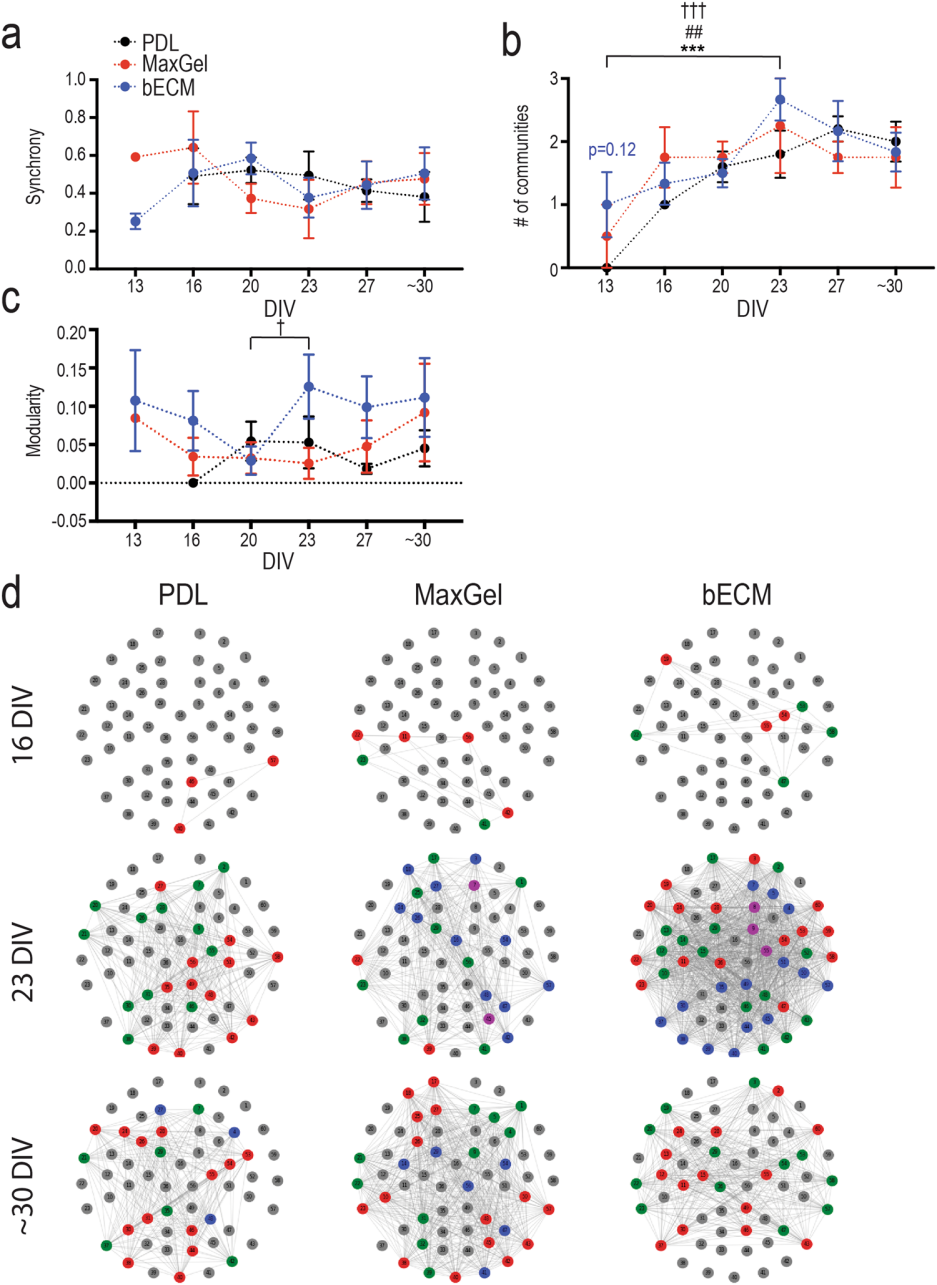
Synchrony and community structure of neural networks formed in the presence and absence of an ECM coating. Line graph summarizes the degree of synchrony between active electrodes on MEA devices (**a**), the number of communities formed (**b**), and the modularity score of these communities (**c**) across ~30 DIV for PDL (black), MaxGel (red), and bECM (blue) experimental conditions. (**d**) Representative MEA plots for PDL (left), MaxGel (middle), and bECM (right) that display 60 electrodes (numbered from 1–60), which are either inactive (grey) or active and part of a community (color) at 16 (top), 23 (middle), and ~30 (bottom) DIV. Data is presented as mean ± SEM for the number of devices in PDL (n = 5), MaxGel (n = 4) and bECM (n = 6) and were analyzed using two-way ANOVA with Tukey’s post hoc test. Statistical significances between time points are at a level of ^#^*p* < 0.05, ^##^*p* < 0.01 and ^###^*p* < 0.001 for PDL (*), MaxGel (^#^), and bECM (^†^).

Synchronized bursting activity is a measure of mature networks *in vitro*^16,19,20,34–39^. In the present study, pairwise comparisons between active electrodes within the 10-minute recording were examined, scoring all possible electrode pairings within the device in terms of specific criteria to establish if the electrode pairs are synchronous or not (see Methods). This comparison generated an averaged synchrony value: 0 having no synchrony to 1 having a high degree of synchrony (Fig. 4a). Synchronous activity developed as early as 13 DIV for both MaxGel (25%, n = 1 out of 4 device) and bECM (50%, n = 3 out of 6 devices). After 16 DIV, the degree of synchronous activity was comparable across all groups.

The development of neuronal networks *in vitro* has been well characterized^16,19,20,34–39^. However, whether these networks can form functionally distinct clusters (i.e., communities) within an *in vitro* system has not been evaluated to date. Here, the graph-theoretic measure of modularity^40^ was used to identify communities within the network of neurons on the MEA (Fig. 4b–d). Modularity is a measure of community structure commonly employed in the analysis of functional brain networks^41^ (Supplementary Fig. 3, see Methods). A ‘community’ is defined as a subset of active electrodes (or nodes) in a MEA that are highly synchronous with each other based on their synchrony scores. These electrodes, however, can also be sparsely connected to electrodes outside the community (Fig. 4d). Representing synchrony scores as weighted links in the graph, multiple iterations of communities are generated to evaluate how synchronized a set of electrodes are relative to the expected synchrony in a random graph. The complexity of the community is determined by the modularity score (Fig. 4c). A score of 0 represents no evidence of community structure different from random probability, whereas a score of 1 represents a high degree of community structure. After identifying the complexity of each community (or modularity) within the MEA, the number of communities established in an *in vitro* system is quantified (Fig. 4b). The number of communities significantly increased from 13 DIV to 23 DIV across all groups, PDL (p < 0.001), MaxGel (p < 0.01), and bECM (p < 0.001), and stabilized by ~30 DIV (Fig. 4b). However, only bECM devices showed increased complexity in the community structure at 23 DIV (relative to 20 DIV, p < 0.05), and trended towards statistical significance compared to age-matched MaxGel devices (p = 0.10, Fig. 4c). Nevertheless, in general ECM-coated devices exhibited non-trivial community structure (i.e., devices with more than one community) as early as 13 DIV and 16 DIV. After 20 DIV, all groups were comparable, with devices characterized by 1 to 3 communities (Fig. 4b). Thus, the inherent properties of network connectivity (i.e., features of spiking and bursting activity) were not largely affected by ECM coating conditions. Rather, the development of synchronized activity and community structures were accelerated and highly reproducible across MEA devices.

**Figure 5:**
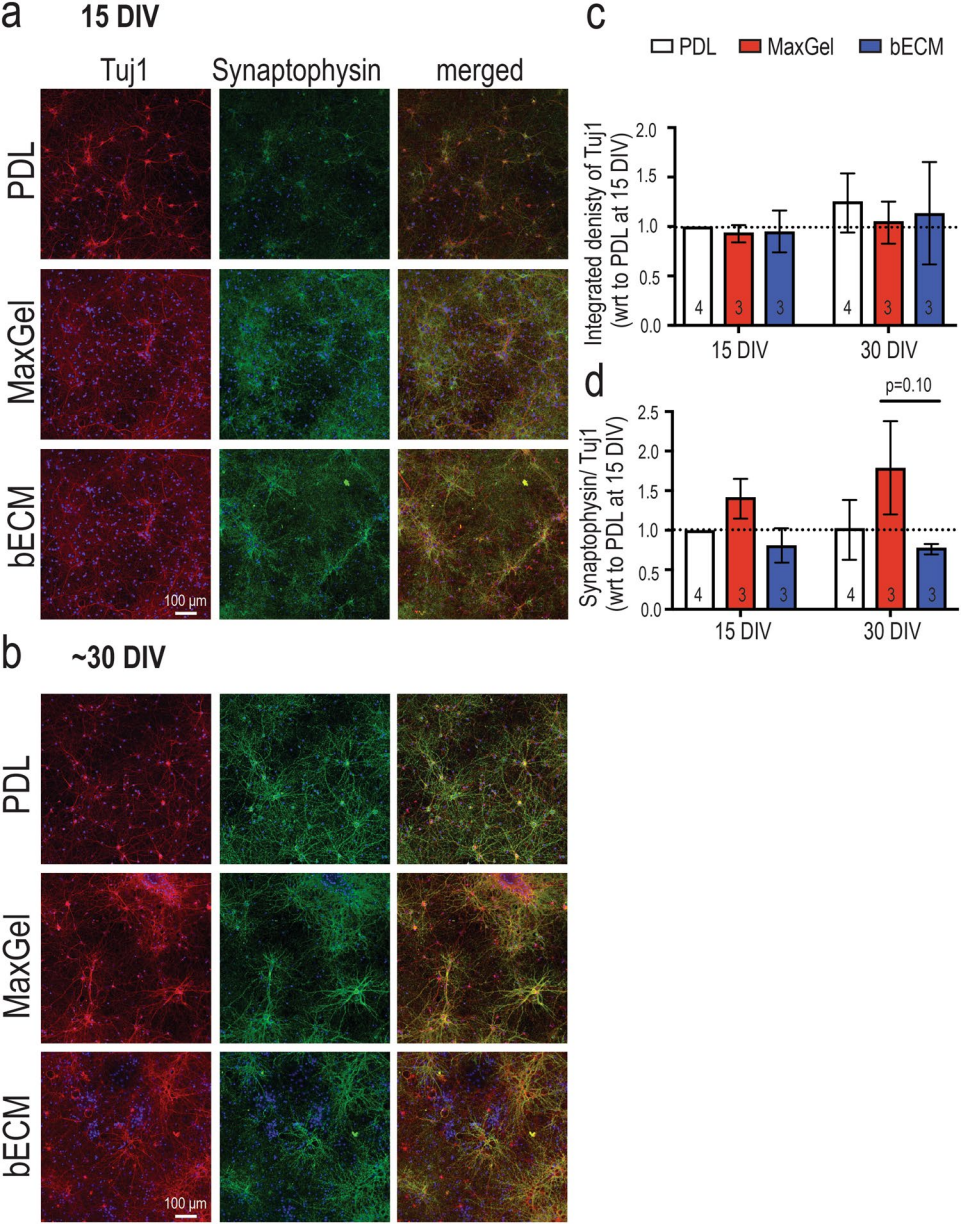
Synaptophysin expression in Tuj1-positive cells grown in the presence and absence of an ECM coating. Representative fluorescent images showing Tuj1-positive neurons (red) and synaptophysin expression (green) at 15 DIV (**a**) and 30 DIV (**b**). Scale bar = 100 μm. Bar graphs summarize the integrated density of Tuj1 (**c**) and synaptophysin normalized to Tuj1 (**d**) per field of view. Data are expressed as a fold change relative to PDL group at 15 DIV, as shown by the dash line, presented as mean ± SEM for the number of cultures indicated at the base of each bar (n = 3–4 biological repeats), and were analyzed using two-way ANOVA with Tukey’s post hoc test.

The acceleration of networks formed *in vitro* from co-cultures grown in the presence of an ECM coating could be attributed to increased synapse formation^42,43^. We asked whether the early onset of synchronous activity in the ECM coating conditions and its effect on neural activity and network formation paralleled changes in synaptic expression. The expression of synaptophysin (normalized to Tuj1 expression), a presynaptic molecular marker, was evaluated at 15 and 30 DIV using immunocytochemistry (Fig. 5a,b). Tuj1 expression remained unaffected by the presence of ECM coatings or across time, although more neurites (or thin processes) extending from Tuj1+ cells were apparent in ECM groups relative to PDL (Fig. 5c). Synaptophysin expression was slightly elevated by ~30 DIV in the MaxGel group (although not significant, p = 0.10), but not in age-matched bECM or PDL cultures (Fig. 5d), which suggests that mechanisms other than synaptophysin expression are involved in the acceleration of networks formed in ECM groups.

### Cellular composition of neuron and glial co-cultures on brain tissue-specific and non-specific ECM coating

**Figure 6:**
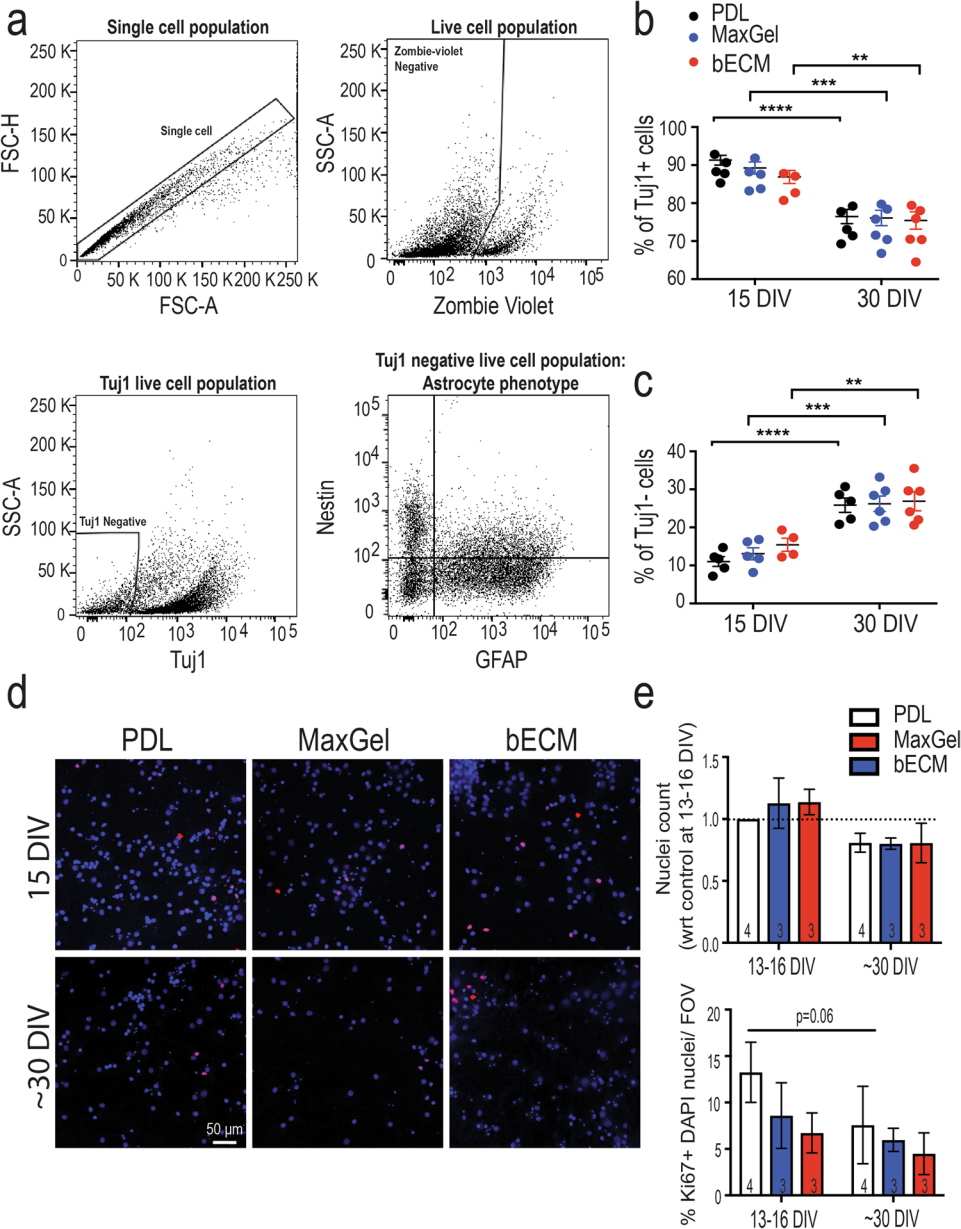
Characterization of cellular composition. Flow cytometry analysis of primary neuron and glial cells co-cultured in the presence or absence of an ECM coating. (**a**) Representative flow cytometry plots illustrate the gating strategy for the co-culture system: single cell population (top left) was gated based on forward-scatter characteristics, Zombie viability dye was used to exclude dead cells (high fluorescence) (top right), and Tuj1 staining was used to identify neuronal (Tuj1+) and glia (Tuj1−) subpopulation of cells (bottom left). The glial subpopulation (Tuj1− cells) was further gated to determine the phenotype of the astrocytic population based on nestin and GFAP expression (bottom right). Scatter plots summarizing the % of Tuj1+ (**b**) and % Tuj1− cells (**c**) from PDL, MaxGel, and bECM culture conditions across time (n = 2–3 cultures from 2 biological repeats). (**d**) Representative fluorescent images of DAPI-stained nuclei (blue) and those positive for the Ki67 proliferative marker (red). Scale bar = 50 μm. (**e**) Bar graph summarizes the total nuclei count (top) and % of Ki67 + nuclei (bottom). Data are mean ± SEM for the number of cultures (n = 3–4 biological repeats) indicated at the base of each bar and were analyzed using two-way ANOVA with Tukey’s post hoc test. Statistical significances between time points are at a level of **p < 0.01, ***p < 0.001, ****p < 0.0001.

To determine whether differences in neural activity were attributable to changes in the cell composition of our long-term co-cultures, flow cytometry was used to assess the ratio between neurons and glia, in addition to characterizing the astrocytic phenotype (see section below). Using standard gating approaches (i.e., doublet exclusion, live cell gating, cell-specific marker gate, see Supplementary Fig. 7), the population of live, single cells (72–77% of total) was identified and analyzed across experimental conditions (PDL, MaxGel and bECM) and time points (15 DIV and 30 DIV) (Fig. 6a). The neuronal marker, Tuj-1, was used to separate the neuronal subpopulation (Tuj1+) from the glial cells (Tuj1−). Neurons represented the majority of cells (~84%-89% at 15 DIV) in our co-culture system and the presence or absence of ECM coating did not affect overall cell count (Fig. 6b). However, analysis of long-term co-cultures showed that the ratio of neurons to glia significantly decreased by up to 15% over time in culture (Fig. 6b). Concurrently, the increase in the glial cell population suggests proliferating glial cells in culture conditions without serum or anti-mitotic agent (Fig. 6c).

To determine whether there was a functional difference in the proliferative capacity of glial cells during long-term cultures and in the presence of an ECM coating, we probed for the presence of Ki67, a nuclear protein that is expressed in proliferating cells during the late G1, S, G2 and M phases^44^. As expected, Ki67 protein was detected in a small subset of cells, which was unaffected by long-term culture or between experimental groups (Fig. 6d,e): PDL (13.25% at 15 DIV and 7.58% at 30 DIV), MaxGel (8.60% and 5.97%) and bECM (6.72% and 4.49%). Thus, the change in the cellular composition of neurons and glia over time in culture is likely due to the basal capacity of glia to proliferate, independent of the ECM coating conditions.

### Phenotypic profiling of astrocytes in the presence and absence of ECM matrices

**Figure 7:**
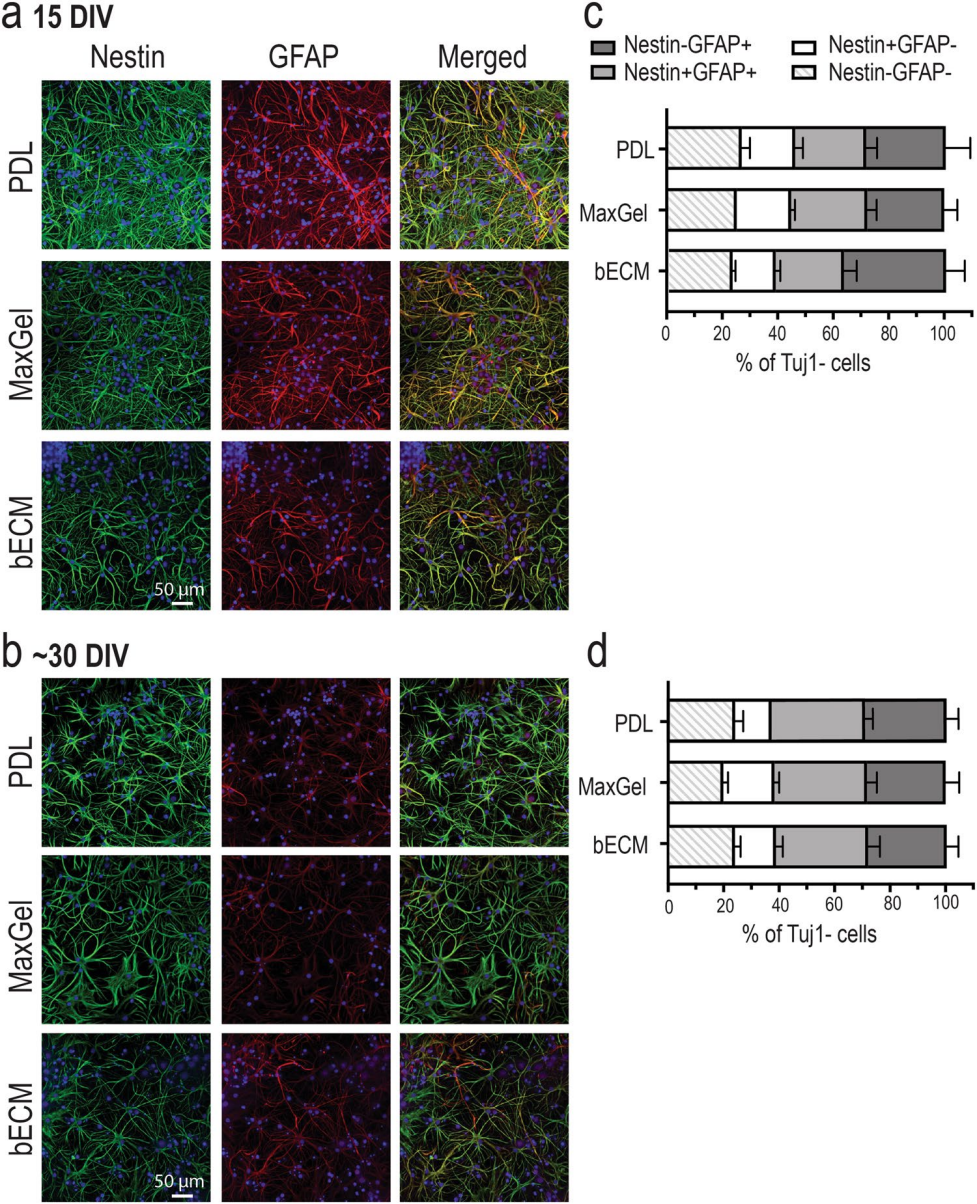
Phenotypic profiling of astrocytes in the presence and absence of an ECM coating. Representative fluorescent images showing a heterogenous population of cells expressing nestin and GFAP at 15 DIV (**a**) and ~30 DIV (**b**). Scale bar = 50 μm. Bar graph summarizes flow cytometry data highlighting the distribution of astrocytic phenotypes (Nestin−GFAP−, Nestin+GFAP−, Nestin+GFAP+, Nestin−GFAP+) at 15 DIV (**c**) and ~30 DIV (**d**). Data are mean ± SEM for the number of cultures (n = 3–4 biological repeats).

Glial reactivity is a response to injury, inflammation or infection, and may have maladaptive effects on *in vitro* neuronal activity^15^. As astrocytes (and possibly low levels of other glial cells) were present in our neuronal cultures^23^, it was important to understand whether the type of ECM coating affects the reactive state of the glial cells present in our cultures. Thus, we assessed the presence of glial fibrillary acidic protein (GFAP) and nestin in astrocytes within our long-term co-cultures with or without tissue-specific ECM coating. An increase in GFAP expression is associated with the maturation of astrocytes in long-term cultures^45–47^. Nestin, an intermediate filament protein, is expressed in immature astrocytes during development, down-regulated in terminally differentiated and mature astrocytes, but re-expressed and upregulated in reactive astrocytes^45–47^. Qualitative observations using immunocytochemistry showed the persistent expression of nestin in cells with multiple and long processes at 15 DIV and 30 DIV (Fig. 7a,b). Furthermore, these cells displayed the co-expression of GFAP and nestin in all three experimental conditions. Because of the heterogenous expression of GFAP and nestin in the co-culture system, flow cytometry was used to quantify the phenotypic state of astrocytes based on the expression of nestin and/or GFAP (Fig. 6a). Immature or reactive astrocytes were identified as Nestin+GFAP− or Nestin+GFAP+, whereas unreactive astrocytes were Nestin−GFAP+ (Fig. 6a, bottom right panel). Among the cells analyzed, more than 55–60% of glial cells (Tuj1−) expressed GFAP, and there was no significant difference in the proportion of astrocytic phenotypes (immature/reactive, unreactive) across experimental conditions or with time in culture (Fig. 7c,d). Collectively, the presence of an ECM coating does not affect the heterogeneity of the astrocytic phenotype (i.e., no shift from unreactive to immature/reactive phenotype) nor its proliferative capacity, despite the accelerated formation of neural networks observed in the ECM groups.

## Discussion

We are ultimately interested in developing *in vitro* neuronal cultures that incorporate key constituents of the CNS, such as ECM and supporting glial cell types, to more accurately recapitulate *in vivo* morphology, physiology, and function. Understanding the effects of ECM on the development and maturation of neuronal co-cultures is an integral part in developing these *in vitro* systems. In the present study, we examined the effects of ECM on long-term co-cultures of neurons and glial cells by comparing neural network formation and maturation in brain tissue-specific ECM (i.e., bECM), non-tissue specific ECM (i.e., MaxGel), and a non-ECM coating (i.e., PDL). We demonstrated that neurons and glia were biocompatible with both tissue-specific and non-tissue specific ECM coating, despite their differences in molecular composition. Furthermore, both ECM coatings had no effect on cellular composition, the proliferative capacity of glial cells, or astrocyte phenotype, the predominant glial cell in long-term cultures. However, neural network formation was accelerated by both bECM and MaxGel as evidenced by synchronized neural activity and development of communities at earlier time points, compared to the absence of ECM coating. Collectively, our study highlights that the addition of ECM coatings in culture systems is a reproducible method that reliably forms neural networks within a shorter time frame and is consistent across devices, reducing the variability that was observed in the control group (or absence of ECM coating). This is particularly important for increasing throughput when evaluating potential neurotoxins and novel therapeutics.

Methods to decellularize tissue and the criteria used to evaluate the efficacy of the decellularization process have been well established for porcine, rat and mouse brains^9,10,12,14,30,48^. In the present study, we used the protocol from Baiguera *et al*.^12^, which itself is a modified version of Ribatti *et al*.^48^, to reproduce the near complete removal of the DNA content (~99.2%, Fig. 1a). Ineffective decellularization of tissue can elicit an immune response^49^; decellularized tissue with dsDNA content less than 50 ng/mg dry weight of tissue is considered to be non-immunogenic^10,14^. The isolated ECM from decellularized rat brain tissue has a preferential banding pattern for high molecular weight proteins (Fig. 1b), consistent with previous studies of decellularized rat and porcine brain tissue^10,14^. Identifying specific ECM molecules present in the decellularized tissue is challenging and limited to immunohistochemistry and immunoassays. Baiguera *et al*.^12^ reported that elastin and sulfated glycosaminoglycans were retained following the decellularization process. Further characterization of the brain ECM was not done in this study, but other studies using rat and porcine decellularized brain tissue have detected myelin, collagen I, collagen III, collagen IV, and laminin^9–11^. Growth factors extracted from the lyophilized ECM included low, but detectable, amounts of VEGF, FGF, BDNF and NGF compared to the native CNS tissue^10,30^. In the present study, the effects of rat brain ECM were compared to MaxGel, a commercial human ECM cocktail derived from basement membrane extract produced from organotypic cell cultures of skin. MaxGel contains collagens, laminin, fibronectin, tenascin, elastin and a number of proteoglycans and glycosaminoglycans^29^. In this study it was used as a reference material to determine whether tissue-specific ECM improved the health and function of neurons and glial cells in long-term culture. While the dsDNA content of MaxGel was similar to decellularized brain tissue, its protein profile showed no bias towards specific molecular weight proteins, suggesting that MaxGel contained proteins not present in the brain.

Previous studies have reported that inefficient decellularization of tissue (e.g., dsDNA content, residual SDS, cellular remnants) can also induce cytotoxicity, or dose-dependent mitogenesis, in addition to eliciting an immune response^10,49,50^. Thus, we evaluated the biocompatibility of the brain tissue-specific ECM and non-tissue specific ECM by examining the cellular composition of the co-cultures, as well as cytotoxicity, the proliferative capacity of the glial cells, and the phenotypes of the astrocytes. We seeded primary neurons in the absence or presence of an ECM coating (bECM or MaxGel) and permitted the growth of glial cells by not adding anti-mitotic agents or serum in the cell culture media. Previous studies have shown that astrocytes or astrocyte-derived molecules are necessary in primary neuronal cultures for synapse formation, maturation, and robust electrophysiological activity *in vitro* compared to pure neuron cultures^26,31–33^. These culture conditions allowed minimal proliferation of glia; approximately 10% of the cells were proliferating by ~30 DIV (Fig. 6d). Using flow cytometry (Fig. 6a), we determined that ~72–77% of the cells in culture were viable, regardless of the presence or absence of ECM coating, and that this viability was maintained over time (assessed at 15 DIV and 30 DIV). However, the shift in the proportion of cells in a mixed co-culture model for all experimental conditions was an effect of long-term culture. While neurons dominated the cell population (~84%-89%) across experimental conditions, a significant decrease (~11–15%) in neurons was observed with time in culture, consistent with previous work^35,51^, which paralleled the increase in the glial cell population (Fig. 6c,d). The proliferative capacity of glial cells under the co-culture conditions was unaffected by the experimental conditions or length of cell culture. In our co-cultures, the glial subpopulation was principally astrocytes: 55–60% of glial cells expressed GFAP in our cell culture system.

To address whether astrocytes were reactive to the ECM coating conditions, we evaluated the astrocytic phenotype based on the expression of two intermediate filaments: (1) GFAP, highly expressed in differentiated and mature astrocytes and upregulated in reactive cells, and (2) nestin, a molecule that is transiently expressed during development, downregulated in mature cells, and upregulated in a reactive state^45–47^. In long-term pure astrocyte and organotypic cultures, the co-expression of nestin and GFAP is detected, although the expression levels depend on the experimental model^46,52^. The persistent expression of nestin in astrocytes appears to be an effect of long-term cultures (both cell and organotypic slices). However, the increased proportion of cells expressing GFAP has been observed in pure astrocyte cultures and not organotypic slice cultures^46,52,53^. In our neuron and astrocyte co-culture, we detected the stable expression of nestin in cells at 15 DIV and 30 DIV using immunocytochemistry (Fig. 7a,b), which is consistent with previous *in vitro* studies^46,52^. Using flow cytometry, the proportion of cells that were immature or reactive (Nestin+GFAP−, Nestin+GFAP+) relative to unreactive (Nestin−GFAP+) remained unchanged across groups over 30 DIV (Fig. 7c,d). This observation could be attributed to the shorter time in culture in our study (up to ~30 DIV versus 8 or 12 weeks), or the use of mixed cell culture versus pure astrocyte culture^46,52,53^. Collectively, the data indicate that ECM coatings (i.e., bECM and MaxGel) are not required to maintain cell viability, do not alter the proliferative capacity of glial cells, and do not affect the astrocytic phenotype in long-term cultures.

In our study, the MEA served as a tool to study and monitor the spontaneous action potential activity of the neurons and the networks that were formed and matured in a 2D *in vitro* model. In a developing culture, the intrinsic capacity of neurons to generate action potentials is important for synaptogenesis^42,43^. Subsequently, neuronal networks are formed *in vitro*, which involves glutamatergic and GABAergic synaptic transmission between excitatory and inhibitory neurons^35–37,42,43^. The onset of sporadic spiking and bursting activity depends on the initial seeding density^38^, which varies across studies from low (e.g. 200–400 cells/mm^2^) to high (e.g., 1000–3000 cells/mm^2^)^16,19,20,34–39^. Regardless of seeding density, the progressive maturation of neural networks was consistent across the abovementioned studies: sporadic action potentials developed into bursting activity, which became synchronized over a period of 28–30 DIV. In the present study, dissociated cortical neurons and glia grown in the presence of either ECM coating (e.g., bECM or MaxGel) accelerated the formation of networks without greatly affecting the inherent network properties (e.g. features of spiking and bursting activity). This is despite the difference in the molecular composition between bECM and MaxGel, suggesting that tissue-relevant ECMs are not required to accelerate the formation and maturation of networks. For instance, by 13 DIV co-cultures grown on bECM or MaxGel coatings showed synchronized bursting activity (Fig. 4a); bursts had more spikes compared to age-matched control cultures (Fig. 3d), and more than one community on MEA devices (Fig. 4b). However, the behavior of the cells grown on ECM was comparable to the control group when networks and communities were formed and matured by 16 DIV and 20 DIV, respectively. Most features of neuronal activity (e.g., overall firing rate, interspike interval, burst duration, and interburst interval) were not affected by the ECM coating conditions (Fig. 3a–f). However, specific features of network activity were dependent on the ECM substrate. By 23 DIV, the number of active electrodes on devices with the brain-specific ECM coating was 2-fold higher relative to non-tissue specific ECM coating (e.g., MaxGel) or in the absence of an ECM coating (e.g., PDL) (Fig. 2c). Conversely, co-cultures on MaxGel ECM coating demonstrated an increase in the rate of bursting activity over ~30 DIV, despite having a comparable number of active electrodes relative to PDL (Fig. 3c). It is important to note the time-dependent and ECM-dependent changes in neural network activity observed were independent of the temporal loss of neurons or increase in glia, as this remained constant across conditions.

While this study showed that both brain tissue-specific and non-specific ECM coatings accelerate the formation of neural activity, it is important to note that neurons and glial cells actively secrete ECM molecules *in vitro* that surround specific regions of a cell to form meshwork structures called perineuronal nets, which can facilitate neural activity^31,34,54–56^. Perineuronal nets are detected and prominent on neurons as early as 2–3 weeks in culture, and degradation of this structure was shown to increase neural activity (i.e., mean firing rate and burst rate) and synaptogenesis in mature hippocampal cultures^54–56^. In the present study, the type of ECM coating (i.e., bECM and MaxGel) had specific effects on network activity. Whether these effects are due to accelerated formation of perineuronal nets or differences in its molecular composition *in vitro* is not clear and outside the scope of this study. However, it will be interesting for future studies to examine whether the bECM- and MaxGel-specific effects on neuronal activity are attributed to differences in the molecular structure of perineuronal nets.

In summary, we demonstrated that neural networks are formed and matured on 3 types of coating conditions: (1) brain tissue-specific ECM; (2) MaxGel, a commercially-available, non-tissue specific ECM; and 3) poly-D-lysine (PDL), a non-ECM coating. Both ECM coatings, independent of the tissue source, accelerated the formation of networks relative to non-ECM PDL, and supported neuronal firing by 13 DIV, which was consistent across all devices within those groups. With the exception of the fact that overall burst rate in the MaxGel cultures was significantly higher than other groups by 30 DIV, the ECM coating had no significant effect on the inherent network properties (e.g. features of spiking and bursting activity) compared to the PDL coating condition. In addition, we demonstrated that complexity of ECM from bECM and MaxGel does not affect cell viability, does not alter the proliferative capacity of glial cells, and does not affect the astrocytic phenotype in long-term cultures. While the biological responses to the matrices examined in this study were not as significantly different as anticipated, the type of characterizations conducted in this study represent a systematic approach to evaluate the interplay between neuronal function, viability, biomarker expression, astrocyte proliferation, and overall culture composition. Collectively, this approach can be applied to evaluating possible effects of other matrices, substrates, culture architecture, and stimulus response on *in vitro* systems developed to recapitulate the brain *in vivo*.

This study highlights the general benefit of incorporating an ECM substrate to augment biological complexity and relevance of 2D *in vitro* neuronal systems, even if the ECM is not compositionally identical to brain ECM. Not only did this modality show reproducibility across multiple MEAs, it accelerated the formation of networks, such as synchrony and community structure, without compromising the intrinsic properties of network connectivity (i.e., features of spiking and bursting activity), the proliferative capacity of glial cells, or changing the phenotypes of astrocytes *in vitro*. Utilizing tissue-relevant ECM coatings for neuronal cultures allows accelerated network formation and maturation within two weeks (rather than four weeks in the absence of ECM), thus providing the means to increase throughput of functional compound screening applications. Future work should be aimed at investigating a variety of ECM formulations to determine their suitability for use in supplementing neuronal cultures in more complex systems (e.g., 3D cultures) in order to maintain long-term viability and neural activity. Identifying culture conditions (e.g., cellular complexity, tissue-specific ECM coating) that improve neuronal network formation and function will be important for developing relevant *in vitro* models that more closely mimic properties of the brain *in vivo* with the goal of ultimately using these systems for evaluating compounds and therapeutics that can influence human health and disease.

## Methods

All procedures on animals adhered to the guidelines and regulations set by Lawrence Livermore National Laboratory, including Institutional Animal Care and Use Committee (IACUC) approval (#220).

### Decellularization of brain tissue

The detergent-enzymatic method was used to decellularize brain tissue^12^. Briefly, 7–9 brain tissues (n = 4 litters) were harvested from postnatal (P3–P6) Sprague-Dawley rat pups (Charles River, Wilmington, MA) and collected in cold PBS. All reagents were from Millipore Sigma (St. Louis, MO) unless otherwise stated. The collected sample was frozen and thawed completely (4X) before incubation with sterile Milli-Q water containing 1% antibiotic and antimycotic solution (72 hr at room temperature). The sample was treated (2X, 60 rpm), as follows: 1% Triton X-100 (60 min), water (30 min), 4% sodium deoxycholate (60 min), water (30 min), 0.2% DNAse I in 1 M NaCl (60 min) and water (30 min). Decellularized tissue sample was lyophilized and stored dry at −80 °C until used for DNA quantification or solubilized for cell culture.

### Solubilization of bECM

Lyophilized bECM was solubilized and enzymatically digested using Pepsin (1 mg/ml in 0.1 M hydrochloric acid) for 24–48 hrs at room temperature^14^. Primary Neuron Basal Medium (PNBM) was added to the solubilized bECM (1:1 ratio), neutralized with 1 M NaOH, and further diluted with basal media to a final stock concentration 1–1.2 mg/ml to match the stock concentration of MaxGel.

### DNA quantification

DNA content of bECM, unprocessed whole brain tissue, and MaxGel, was extracted using the PureLink Genomic DNA Mini kit (Thermo Scientific, Franklin, MA). Briefly, samples were digested using proteinase K and RNase A and followed by DNA spin columns to bind and elute the genomic DNA. Extracted DNA was quantified using Quant-iT PicoGreen dsDNA assay kit (Thermo Scientific) using three dilutions (1:20, 1:50, and 1:100) done in duplicate.

### Protein gel electrophoresis

All reagents were from Thermo Fisher Scientific, unless otherwise stated. Lyophilized bECM and MaxGel protein (50 µg) from stock were denatured (70 °C for 10 min) in NuPAGE LDS sample buffer with NuPAGE reducing agent. Denatured samples and unstained Mark 12 protein ladder were loaded onto a 4–12% Bis-Tris protein mini gel and ran in NuPAGE MOPS SDS buffer containing NuPAGE antioxidant (75 min, 200 V). The protein gel was stained with SYPRO ruby protein gel stain, then washed with 10% methanol, 7% glacial acetic acid solution, and rinsed with deionized (DI) water, before being imaged at 600 nm with an Odyssey® FC imaging system (LI-COR Biotechnology, Lincoln, NE).

### Multielectrode array (MEA) fabrication

Fabrication of the MEA was described in our previous work^23,25^. Briefly, MEAs contain 60 platinum electrodes (50 μm in diameter) spread evenly over 1.8 mm^2^ on a glass substrate. After fabrication, a polystyrene cylinder was affixed over the MEA to enable cell culturing. ZIF connectors were added to the device for electrical connections and electrodes were plated with platinum black. Impedance measurements were taken prior to seeding and ranged from ~50–250 kΩ at 1 kHz. MEAs were sterilized with 70% ethanol (20 min) and rinsed (3X) with sterile water before use.

### ECM coating and cell culture

MEAs or wells in a 96-flat bottom-well plate were coated with 0.1 mg/mL poly-D-lysine (Millipore-Sigma), washed with sterile DI water (4X), and air-dried before use. PDL-coated MEA and wells were then left uncoated or coated with MaxGel (Millipore-Sigma) or bECM at a 1:25 dilution from stock in neuron media consisting of PNBM supplemented with 2 mM L-glutamine, 50 μg/mL gentamicin, 37 ng/ml amphotericin, and 2% NSF-1 for 2 hr (37 °C, 5% CO_2_). Primary rat embryonic cortical neurons (Lonza, Walkersville, MD) were seeded on devices and wells at a density of 1200 cells/mm^2^ and maintained in a humidified incubator (37 °C, 5% CO_2_). Media change (50%) occurred every 3–4 days. Custom device caps, made from a polytetrafluoroethylene (PTFE) housing and a fluorinated ethylene-propylene (FEP) membrane (ALA Scientific, Farmingdale, NY), were used to maintain sterility and to allow for gas exchange.

### Electrophysiology recording and processing

A multi-channel recording system (AlphaLab SNR, Alpha Omega, Alpharetta, GA) was used to record electrophysiology activity for 10 minutes at a sampling frequency of 22.3 kHz and bandpass filtered between 268–8036 Hz. Devices were placed within a 5% CO_2_-regulated chamber on a heated stage at 37 °C. Raw spike data was converted to Matlab files, and imported into RStudio software (Boston, MA) for data processing and analysis using an in-house custom R package, which removed noisy electrodes (>4000 spikes), noise artifacts seen across all channels, and silent electrodes (<10 spikes). An action potential spike was defined by a lower limit threshold, set at 5x the standard deviation of baseline noise, for each electrode. Feature analyses for spikes and bursts were calculated based on previous work^57^, and include: firing rate, interspike interval (ISI), burst per minute, burst duration, percentage of spikes within bursts, and interburst interval (IBI). Burst parameters, defined previously^23,25^, include: maximum beginning ISI of 0.1 sec, maximum end ISI of 0.2 sec, minimum IBI of 0.5 sec, minimum burst duration of 0.05 sec, and minimum number of spikes per burst of 10.

### Synchrony and network analysis

Synchrony was assessed using SPIKE-distance^58^. The package *PySpike*^59^ computed the SPIKE-distance between every pair of active electrodes in a MEA (Supplementary Fig. 3a,b). Since two spike trains with uniformly random activity may exhibit relatively high synchrony merely due to randomness, the SPIKE-distance from recorded data was normalized to random samples generated computationally. That is, for a pair of electrodes with n1 and n2 spikes, random spike trains were generated by sampling n1 and n2 points uniformly at random in a 10-minute timeline. This process was repeated 1,000 times, and the average normalized score is reported. Herein, synchrony is defined as SPIKE-distance values subtracted from 1 (denoted as 1-SPIKE distance), so that synchrony values close to 1 represent a high degree of synchrony, and values close to 0 represent asynchrony.

Community structure on the MEA was examined by graph-theoretic analysis, using the Louvain algorithm for modularity maximization^40,60^. In the analysis, electrodes are modeled as ‘nodes’, and weighted ‘edges’ or links between every pair of active electrodes were assigned with weight equal to the synchrony value. Only links with synchrony below 0.10 were filtered out, making the graphs similar to a clique in structure. This relatively low threshold preserved most of the synchrony data, which was desirable, since the purpose of the analysis was community detection—instead of, for instance, measuring small-worldness, density, and other graph structural properties. Nodes and edges on the MEA (shown in Fig. 4) were analyzed using the modularity function^40^. Modularity measures the complexity of the community structure in a graph by determining how much more connected the nodes in a community are compared to a random graph. A modularity score is determined: 0, having no evidence of community structure different from chance, to 1 having a high degree of community structure. Using the Louvain method (https://github.com/taynaud/python-louvain), nodes are merged into possible communities, their modularity scores computed, and recomputed as new merged communities are generated until there is no merge operation that increases the score—i.e., modularity is maximized. At the end, electrodes in the same community are more densely connected to each other than to those in other communities (Supplementary Fig. 3c). Communities were detected for all devices and DIVs, and a representative device is shown in supplementary figures for PDL (Supplementary Fig. 4), MaxGel (Supplementary Fig. 5), and bECM (Supplementary Fig. 6).

### Immunocytochemistry

Primary cultures (devices and 96-well plate) were fixed with 4% paraformaldehyde (PFA, 20 min), washed in PBS (3X), blocked with 10% goat serum (1 hr), and incubated overnight at 4 °C with primary antibodies in a saponin-based 1X perm/wash solution (BD Biosciences, San Jose, CA). Cells were labeled with anti-class III beta-tubulin (Tuj1, chicken, 1:200, Neuromics, Edina, MN), anti-nestin (chicken, 1:200, Neuromics), anti-GFAP (mouse, 1:1000, Millipore-Sigma), anti-synaptophysin (rabbit, 1:250, Abcam, Cambridge, MA), or anti-Ki67 (mouse, 1:10, BD Biosciences). Cells were washed with PBS (3X) and incubated (1 hr in the dark, room temperature) with a secondary antibody (Life Technologies, Eugene, OR) at 1:500 or 1:1000 dilutions in 1X perm/wash solution: Alexa Fluor 488-conjugated goat anti-mouse, Alexa Fluor 594-conjugated goat anti-rabbit, Alexa Fluor 647-conjugated goat anti-rabbit. For nuclear staining, cells were incubated (10 min) in 4′-6-diamidino-2-phenylindole (DAPI; 1:3000; ThermoFisher). Samples were stored in PBS (dark, 4 °C) before imaging using a LSM700 confocal microscope (Carl Zeiss Microscopy, Thornwood, NY) and quantified using Fiji software (https://fiji.sc/#; National Institutes of Health, Bethesda, MD).

### Flow cytometry

Cells were detached with 0.1% trypsin-EDTA, washed, and triturated to a single cell suspension in 10% FBS in PBS. Cells were pelleted and washed with PBS to remove all residual FBS. All reagents were from BD Biosciences, unless otherwise stated. For cell viability staining, cells were incubated (15 min) with Zombie Violet fixable viability dye (1:1000, Biolegend, San Diego, CA). For antibody staining, cells were fixed with 4% PFA (20 min) and washed and resuspended in 1X perm/wash solution. Neurons were characterized using Alexa Fluor 647 mouse anti-Tuj1 (1:25), and astrocytes with Alexa Fluor 488 mouse anti-GFAP (1:100) and mouse anti-Nestin (1:50, Neuromics), conjugated to PE-Cy7 using the Lightning-Link R-PE-Cy7 Kit (Novus Biologicals, Littleton, CO) according to manufacturer’s instructions. Samples were washed and resuspended in FBS stain buffer for immediate measurement. Samples and compensation controls were measured using a BD Aria Fusion instrument and analyzed using FlowJo version 10 software (Tree Star, Inc., Ashland, OR). Single, live cells were identified according to forward- and side-scatter characteristics and low Zombie-Violet fluorescence intensity (Supplementary Fig. 7a). Thresholds for individual fluorophore gates were set to estimated background level as informed by fluorescence-minus-one controls prepared from subsets of all samples, pooled prior to staining (Supplementary Fig. 7b–d).

### Statistics

Quantified data are expressed as mean ± SEM for the number of replicates indicated. For DNA, immunocytochemistry, cell staining, and electrophysiological data, the statistical significance was analyzed in GraphPad version 7 (GraphPad Software, San Diego, CA) using unpaired t-test or two-way ANOVA with and without repeated measures with Tukey’s post-hoc analysis.

## Supplementary information

Supplementary Figures
