# Supplementary material for: Tissue-specific extracellular matrix accelerates the formation of neural networks and communities in a neuron-glia co-culture on a multi-electrode array
Source: Sci Rep. 2019 Mar 11;9:4159. doi: 10.1038/s41598-019-40128-1 (PMC6411890; doi:10.1038/s41598-019-40128-1)
Supplement: Supplementary file 1 — Supplementary Figures [file 41598_2019_40128_MOESM1_ESM.pdf]

Tissue-specific extracellular matrix accelerates the formation of neural networks and communities in a neuron-glia co-culture on a multi-electrode array

Doris Lam[1], Heather A. Enright[1], Jose Cadena[2], Sandra K. G. Peters[1], Ana Paula Sales[2], Joanne J. Osburn[1], David A. Soscia[2], Kristen S. Kulp[1], Elizabeth K. Wheeler[2], and Nicholas O. Fischer[1,*]

1. Physical and Life Sciences Directorate, Lawrence Livermore National Laboratory, Livermore, CA, USA
2. Engineering Directorate, Lawrence Livermore National Laboratory, Livermore, CA, USA

Supplementary Figures

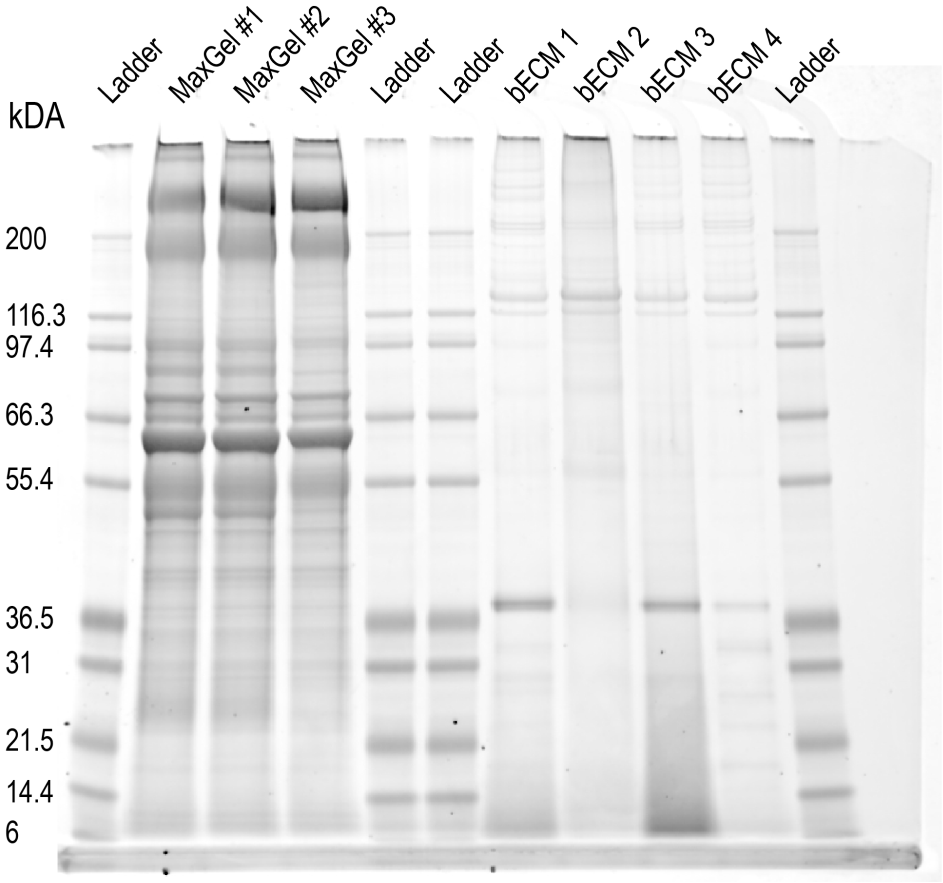

Supplementary Figure 1. Gel electrophoresis of MaxGel and bECM. Full length gel shows multiple bands spanning from low to high molecular weights for MaxGel samples (from different commercial lot numbers) compared to bands shown in bECM samples (also shown in Figure 1).

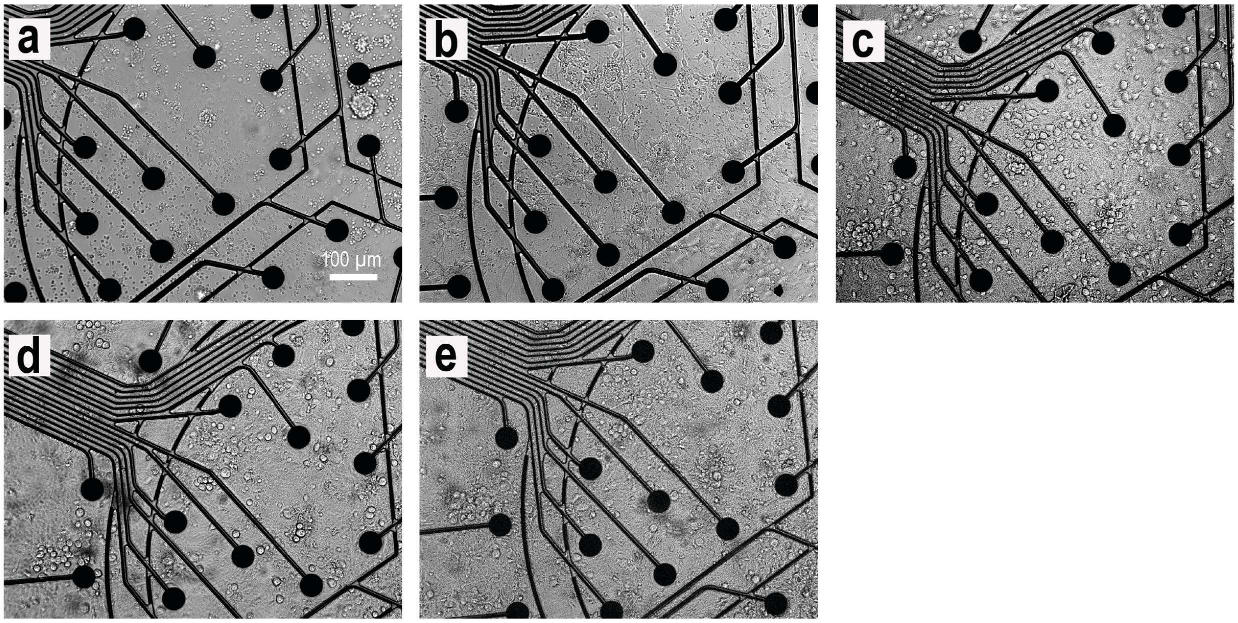

Supplementary Figure 2. Long-term neuron and glial co-cultures grown on bECM-coated MEA devices. Representative brightfield images show neuron and glial cells co-cultured on bECM-coated MEA devices for 1 (a), 7 (b), 14 (c), 28 (d), and 32 (e) DIV. Scale bar= 100 µm.

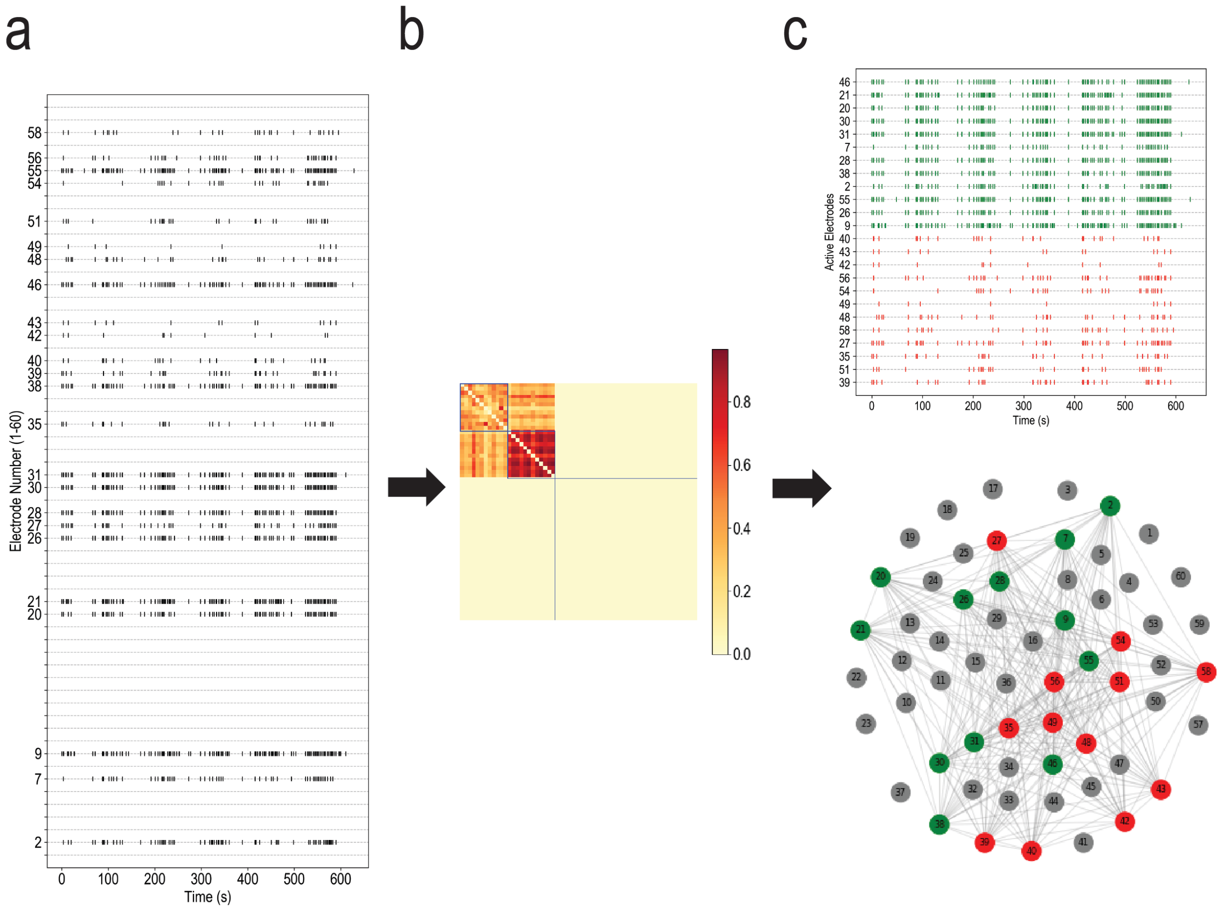

Supplementary Figure 3. Processing neural network data for synchrony and community structures. From one MEA device, a representative raster plot is generated displaying all 60 electrodes in the 10-minute recording, and only those numbered were active (a). Each hash line indicates an action potential. The matrix summarizes the synchrony scores computed from 0 (no synchrony) to 1 (highly synchronous) between every pair of active electrodes on the device (b). In the functional network, nodes represent active electrodes and the link between two electrodes is weighted equal to their synchrony score. (c) Communities in the functional network were discovered via modularity maximization. For the recording in this figure, two communities were identified. We show the spike train sorted and color-coded by community (top) and the spatial locations of the nodes (electrodes) of the graph in the multi-electrode array (bottom).

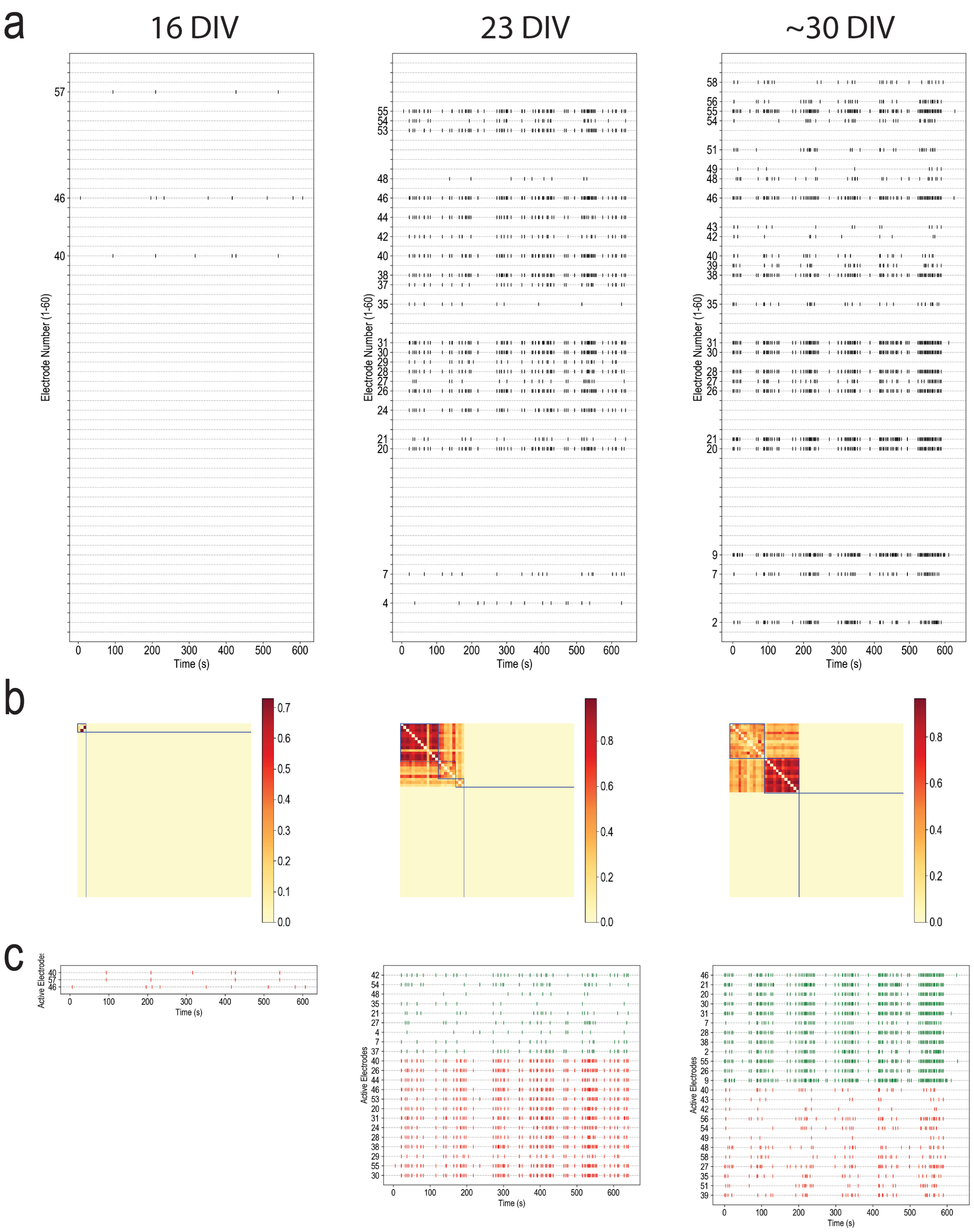

Supplementary Figure 4. Temporal progression of synchronous and asynchronous neural networks over 30 DIV for co-cultures grown in the absence of ECM coating. The development of neural networks in a representative device without ECM coating (PDL) over selected time points: 16 (left), 23 (middle), and

right ~30 DIV. (a) Representative raster plots displaying all 60 electrodes in the 10-minute recording, and only those numbered were active. Each hash line indicates an action potential. (b) Matrices summarize the synchrony scores computed for the device at each time point and were used to detect the number of communities (c). Very sparse activity is observed in 16 DIV, leading to a single community structure in the functional network. From 23 DIV onwards, community analysis reveals two distinct groups of electrodes (indicated by red and green).

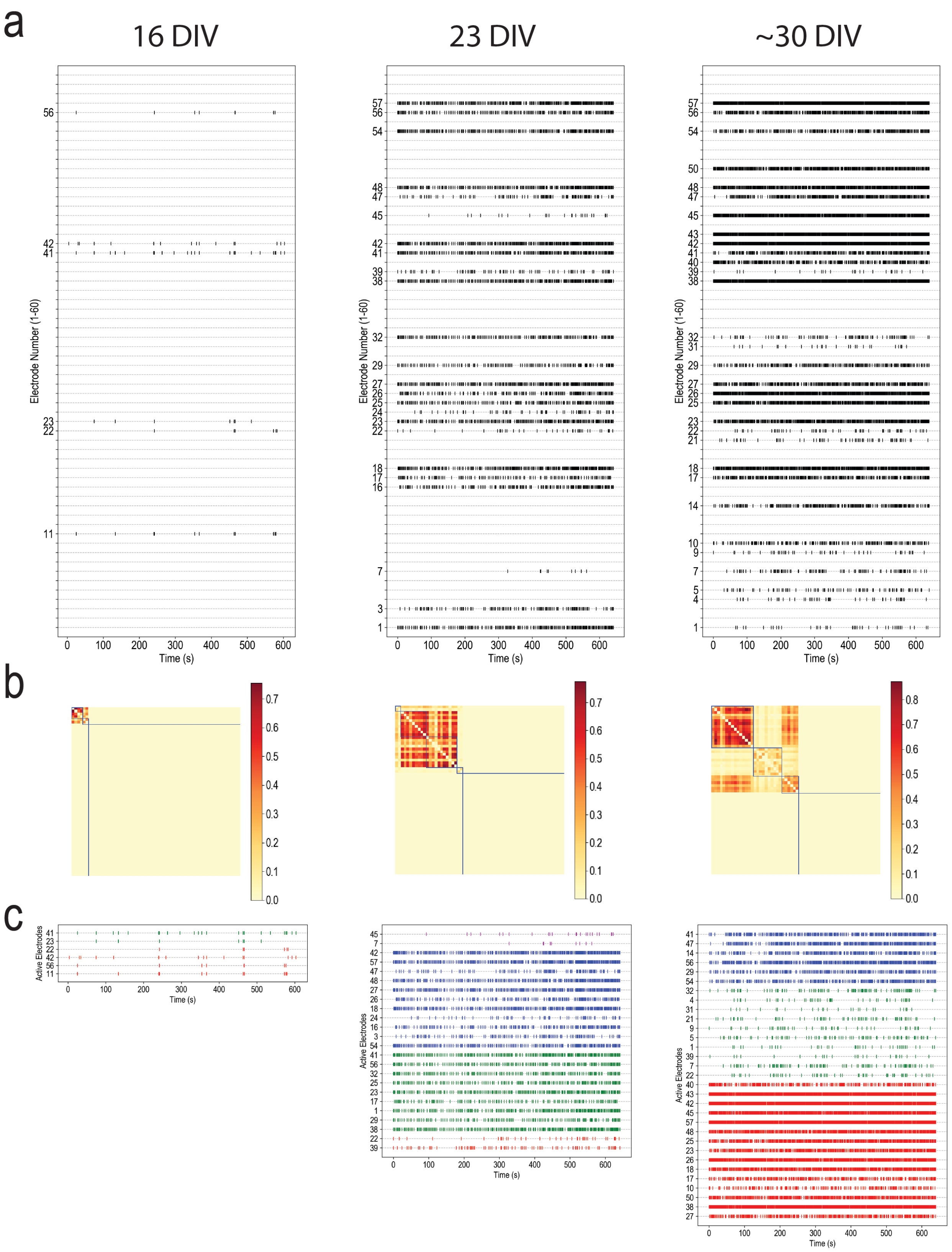

a
16 DIV
23 DIV
~30 DIV
Electrode Number (1-60)
Time (s)
b
c
Active Electrodes
Time (s)

Supplementary Figure 5. Temporal progression of synchronous and asynchronous neural networks over 30 DIV for co-cultures grown on MaxGel coating. The development of neural networks in a representative device with MaxGel ECM coating over selected time points: 16 (left), 23 (middle), and right ~30 DIV. Raster plots (a), matrices (b), and communities (c) are as described in Supplementary Figure 3. Communities are present in MaxGel devices from 16 DIV, with more communities (indicated by red, blue and green) developing over time.

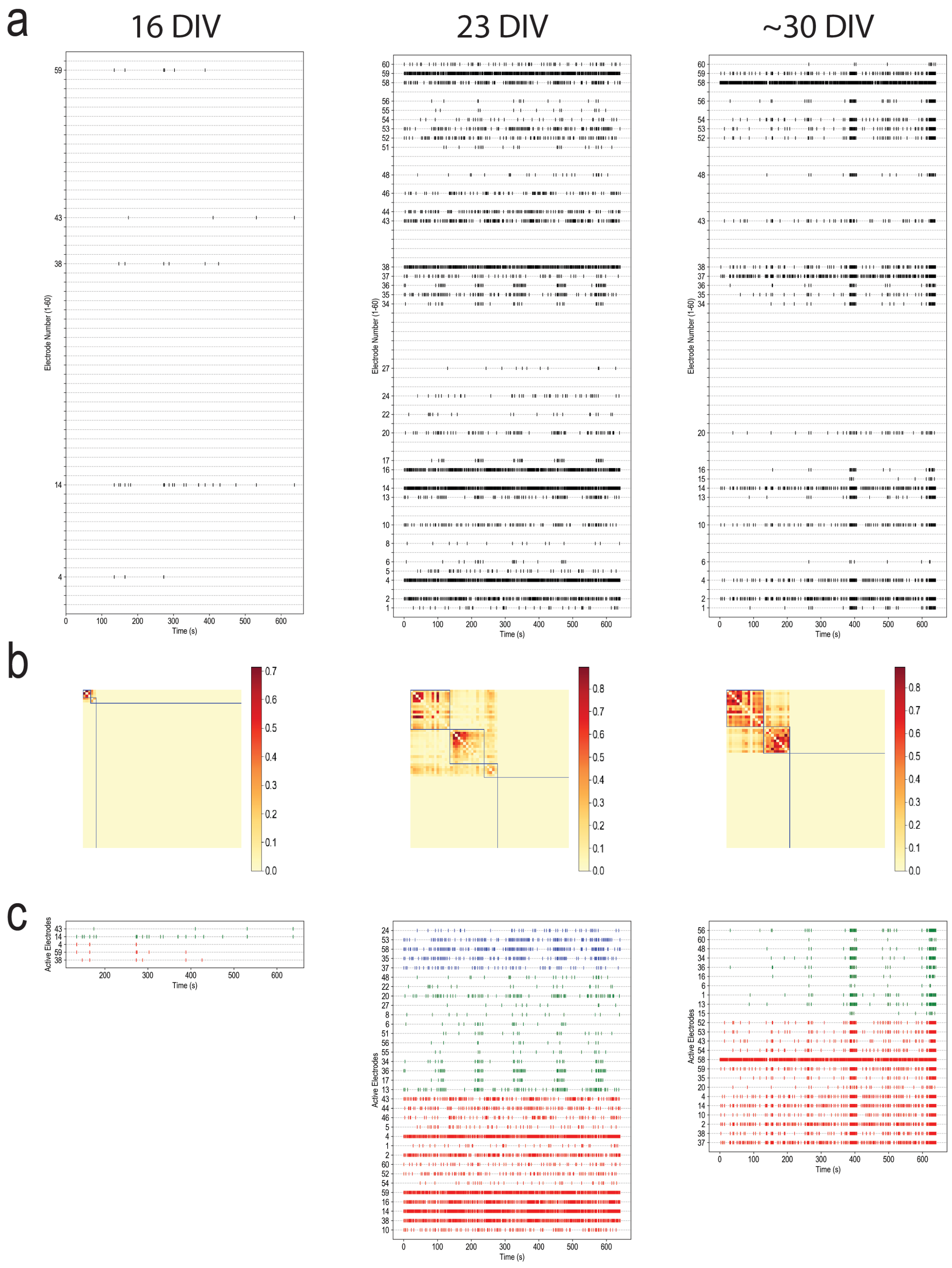

Supplementary Figure 6. Temporal progression of synchronous and asynchronous neural networks over 30 DIV for co-cultures grown on bECM coating. The development of neural networks in a

representative device with bECM coating over selected time points: 16 (left), 23 (middle), and right ~30 DIV. Raster plots (a), matrices (b), and communities (c) are as described in Supplementary Figure 3. Communities are present in bECM devices from 16 DIV. A surge of electrode activity and community formation was observed in 23 DIV, but, by 30 DIV, the device settled into two community groups.

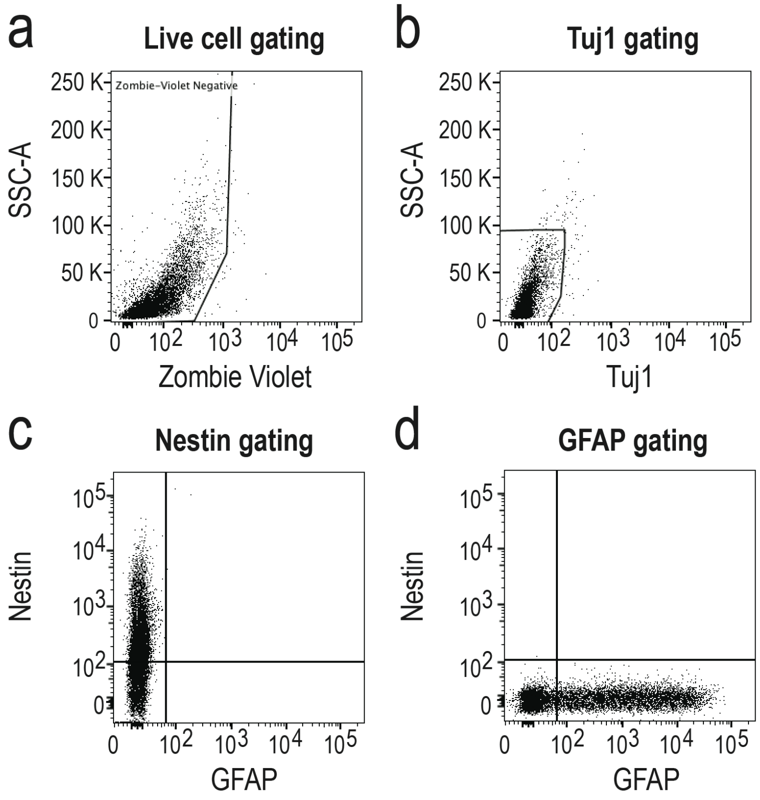

Supplementary Figure 7. Gating approach for co-culture system flow cytometry samples. Single cells were identified according to forward- and side-scatter characteristics (as described in manuscript text). Thresholds for individual fluorophore gates: Zombie Violet (a), Tuj1 (b), nestin (c) and GFAP (d), were set to estimated background level as informed by fluorescence-minus-one controls prepared from subsets of all samples, pooled prior to staining.
